# Health Disparities and Cardiovascular Disease

**DOI:** 10.3390/healthcare8010065

**Published:** 2020-03-22

**Authors:** Ava Niakouei, Minoo Tehrani, Lawrence Fulton

**Affiliations:** 1 College of Liberal Arts & Sciences, University of North Carolina Charlotte, Charlotte, NC 28223, USA; aniakoue@uncc.edu; 2 Mario J. Gabelli School of Business, Roger Williams University, Bristol, RI 02809, USA; mtehrani@rwu.edu; 3 Health Administration, Texas State University, San Marcos, TX 78666, USA

**Keywords:** cardiovascular disease, smoking, drinking, underserved, health disparities

## Abstract

The number one leading cause of death in 2017 for Americans was cardiovascular disease (CVD), and health disparities can exacerbate risks. This study evaluates the 2018 Behavioral Risk Factor Surveillance System (BRFSS) (*n* = 437,436) to estimate population risks for behavioral, socio-economic, psychological, and biological factors. A general linear model with a quasi-binomial link function indicated higher risks for the following groups: smokers (odds ratio, OR = 0.688), individuals with higher body mass index scores (OR = 1.023), persons unable to work (OR = 2.683), individuals with depression (OR = 1.505), workers who missed more days due to mental issues (OR = 1.12), the elderly, males (OR = 1.954), those in race categories “indigenous Americans, Alaskan non-Hispanics”, “Black Hispanics,” or “other, non-Hispanic,” and individuals with lower income. Surprisingly, increased consumption of alcohol was not found to be a risk factor as in other studies. Additional study of alcohol risk factors is needed. Further, Black non-Hispanics were associated with lower rates of CVD/MI (myocardial infarction), a finding that is supported by recent evidence of more unhealthy behaviors in other races. The results of this study highlight 2018 CVD/MI disparities based on the BRFSS and suggest the need for additional policy interventions including education and providing increased access to health care for the disadvantaged. The principles of beneficence and justice require policy interventions such as these.

## 1. Introduction

The number one leading cause of death in 2017 for Americans was cardiovascular disease. Cardiovascular disease (CVD) is the leading cause of death globally, taking about 17.9 million lives yearly [1]. Heart disease took the lives of 647,457 people in 2017, and the projected numbers are assumed to increase with the next coming years [2]. Mortality from cardiovascular disease has accounted for 31% of the total deaths in the world [1] and 1 in 4 deaths in the United States [3]. 

Due to the prevalence of heart disease around the world, measures should be taken to identify individuals with CVD risk factors, particularly those risk factors that might be modified to reduce mortality. Included in that grouping are behaviors such as smoking, body mass index, lack of physical activity, and excessive alcohol consumption [4]. Behavioral risk factors are often related to socio-economic, psychological, and biological factors, so these factors must be investigated as well. 

There are many socio-economic factors that affect CVD mortality rates. Lower socio-economic status has been linked to the development of cardiovascular disease [5,6]. Disparities in health care that favor racial and ethnic majority groups are particularly notable for CVD and cancer [7]. Lower-income individuals also have higher health risks associated with CVD, and rural residents have less access to care and more risk factors [8]. For example, food deserts, generally poor areas such as inner cities where individuals have limited access to healthy retail food stores, have been associated with childhood obesity, a known risk factor for CVD [9]. A study in New York City found that the population suffering from higher rates of chronic conditions like obesity and diabetes were black population living in food desert neighborhoods [10]. 

Behavioral and socio-economic disparities associated with CVD are two important considerations for identifying and reducing morbidity and mortality risks. But other factors, including biological and psychological, must be considered as well. For example, aging has biological impacts associated with heart disease [11] along with race [12], while psychological risk factors include loneliness and isolation [13].

### 1.1. Behavioral Factors

Behavioral factors have a significant impact on health, including CVD/MI (myocardial infarction, also known as a heart attack) [14]. Addiction is one social determinant of health that is associated with social deprivation [14]. Previous studies have shown that Blacks, Hispanics, Asians, and Indigenous Americans are at a higher risk of addiction [14]. Addiction to tobacco, drugs, alcohol, behavior, or food can severely weaken the body and increase the number of premature deaths, which is also related to low income and unemployment or harsh economic status [14]. Lack of exercise is associated with both increased body mass index (BMI) and CVD/MI [14]. This study incorporates self-reported smoking, alcohol, other tobacco use, BMI, and physical activity as proxies for behavioral factors that may be associated with CVD/MI. 

### 1.2. Socio-Economics

A 2011 study performed by Kucharska-Newton [15] in the United States found that the increase of the probability of heart disease is associated with low-income individuals and neighborhoods where these individuals engage in behaviors such as excessive drinking and smoking. These socio-economic factors affect behaviors synergistically and may be used to predict the risk of cardiovascular disease. In addition, the study indicates that a $10,000 increase in the median income reduces death associated with the cardiovascular disease by 10%. Residents in low-income areas are less likely to receive proper health care for the CVD. This issue might be due to the fact that low-income areas are less likely to be able to afford expensive procedures dealing with heart disease [16]. Even if they were able to afford the procedures, they may not be able to keep up with the needed follow-up and expensive prescriptions. This issue is one of the major contributors to the increased mortality rate for those who have a lower income.

Mortality differences in patients who have a lower income are also due to disparities in the standards of provided health care and decreased access to quality care for the socially disadvantaged. This discrepancy has been shown to lead to an increase in heart failure and hospital readmission rates in the United States [5]. There is also the fact that lower-income patients are more likely to have fewer yearly medical checkups, which can also lead to a higher occurrence of CVD.

The next socio-economic factor regarding cardiovascular disease is educational attainment. A study performed by Woodward (2015) in Australia and New Zealand indicates those who only have primary education are at higher risk of CVD than the ones with tertiary education [17]. The correlation between less education and increased cardiovascular disease might be attributable to behavioral and biological risk factors as well. These factors include smoking, obesity, physical inactivity, and hypertension [5]. In addition, there is a strong correlation between education and health literacy. Individuals who have poor health literacy are less likely to be as compliant with the prescriptions prescribed. This study evaluates educational attainment as a risk factor for CVD.

Unemployment has been seen to increase the risk of cardiovascular events [5]. The detrimental effects of unemployment may be driven by the loss of the job itself. There is also the theory that poor health results in the loss of employment [5]. An explanation for the impact of unemployment on cardiovascular disease could be an accumulation of stress that could lead to overuse of alcohol and tobacco. Employment status is another factor used in this study.

The last socio-economic factor that can increase the risk of cardiovascular disease is environmental factors. A 2015 study by Dubowitz using data on food-purchasing practices, dietary intake, height, and weight from the primary food shopper in randomly selected households illustrates the many effects of living in a disadvantaged region [18]. Obvious issues associated with such regions include lower income, lower educational attainment, and higher unemployment; however, less obvious is that these factors may correlate with the presence of CVD. To account for this factor, geographic location (e.g., inner city) is used in this study. 

### 1.3. Biological Factors

Race and age are biological factors associated with CVD [11,12]. Though age is a common health disparity when it comes to the topic of heart disease, race and ethnicity are important considerations as well. Black and Hispanic communities have experienced many health disparities, including a lower status of health care, lack of access to health insurance, increased use of tobacco, and obesity, among other factors. Being subjected to copious degree of racism and discrimination can cause stress, which in turn can increase the risk of heart disease [14]. This study includes age and race as well as gender to evaluate biological risk factors. 

### 1.4. Psychological Factors

Individuals that live and work in low socio-economic status environments may feel the effects of diminished self-esteem, lower sense of control, and a reduced ability to be productive [5]. The living environment has been associated with the development of a pessimistic outlook on life and the resultant negative psychological effects [13]. These psychological conditions can lead to poor choices and contribute to CVD risk factors. For this reason, the study includes a variable that assesses individual psychological status, specifically depression. 

### 1.5. Research Question 

This study examines socio-economic, psychological, biological, and behavioral factors that are associated with CVD using the Behavioral Risk Factor Surveillance System of the Centers for Disease Control and Prevention (CDC) [19]. The research questions are straightforward: What behavioral, socio-economic, psychological, and biological variables are associated with CVD based on the 2018 Behavioral Risk Factor Surveillance System (BRFSS)? The exact relative risks are reported for inclusion. 

This study is significant since it updates previous research with the most recent data and investigates multiple variables to assess their relative risks. Hypothetically, one might expect a reduction in disparities given the passing of the Affordable Care Act and the focus on health disparities. The study is based on anonymous, publicly available data from the CDC. No institutional review board was required. 

## 2. Materials and Methods 

The 2018 BRFSS provided the data for this study. This dataset includes 437,467 observations and weights. Applying the weights estimates the entire population. Data from the BRFSS are freely available from the CDC [19]. Analysis of the data was conducted both with and without weights. 

The dependent variable of interest was a calculated variable from the dataset. The variable was MICHD and defined as “Respondents that have ever reported having coronary heart disease (CVD) or myocardial infarction (MI) [20].” Responses were dichotomous: (0 = No, 1 = Yes). Only 6.89% of the weighted observations were “Yes,” resulting in imbalanced data. 

The behavioral independent variables of interest included variables that evaluate CVD as a function of behavioral, socio-economic, psychological, and biological predictors. Behavioral variables included smoking, other use of tobacco (dichotomous), and drinks per week (quantitative). Smoking was defined by the categorical variable SMOKER3 from the BRFSS: “Four-level smoker status: Everyday smoker, Someday smoker, Former smoker, Non-smoker [20].” Use of other tobacco products was defined by the variable USENOW3: “Do you currently use chewing tobacco, snuff, or snus every day, some days, or not at all?” [20]. This variable was recoded to dichotomous, as there were few observations of individuals who actually used snuff or chewing tobacco. Drinks per week was a calculated variable from the variable DROCDY3: “Drink occasions per day [20].” Body mass index (BMI) was a quantitative variable generated from BMI5 in the BRFSS. Physical activity was also quantitative and indicates “Adults who reported doing physical activity or exercise during the past 30 days other than their regular job. [20]” 

The socio-economic variables included income (categorical), education (categorical), employment (categorical), and urban/rural status (dichotomous). Variable definitions for income, education, and employment are in Table 1, Table 2 and Table 3, respectively. Urban/rural status was defined by the variable METSTAT: “Metropolitan Status” [20]. 

Psychological variables included depression (dichotomous) and the number of days in the last month where mental issues affected activities (quantitative). Depression was defined from variable ADDEPEV2: “(Ever told) you have a depressive disorder (including depression, major depression, dysthymia, or minor depression)? [20].” Days lost due to mental issues was based on variable MENTHLTH: “Now thinking about your mental health, which includes stress, depression, and problems with emotions, for how many days during the past 30 days was your mental health not good? [20]”. The biological variables included age (categorical groups, see Table 4), gender codes as (0 = female, 1 = male), and race (categorical, see Table 5).

The model (in blocks) follows. CVD/MI is a function of behavioral, socio-economic, psychological, and biological variables. The researchers expected to find disparities in all areas. The method for evaluating these variables was the application of a general linear model (GLM) with a quasi-binomial error term. The quasi-binomial distribution does not check for integer status (appropriate for weighted surveys where integers might become fractions) and accounts for variance in the data not explained by a binomial alone [21]. The distribution is often used for surveys which are weighted where the weights may make counts non-integer. This approach is identical to that of logistic regression where the outcome may not be integer. Equation (1) is the quasi-binomial formula, where *p* is the probability of CVD/MI, *N* is the number of weighted observations, *k* is the number of successes (perhaps non-integer due to weighting), and *ϕ* is the additional variance not accounted for by the binomial distribution. All analyses were run in R Statistical Software [22]. The survey package in R was used for complex weighting [23].

(1) $$P\left(X=k\right)=\left(\begin{array}{c} N\\ k \end{array}\right)p{\left(p+k\phi \right)}^{k-1}{\left(1-p-k\phi \right)}^{n-k}$$

## 3. Results

### 3.1. Descriptive Statistics

Only 2% of observations were missing from the complete dataset, so simple imputation (mode for categorical and mean for quantitative) was used. All data were made complete for 437,436 unweighted observations. Data were inspected by validating frequencies in the BRFSS codebook and descriptive statistics. The clean and fully populated data were then used in all analyses.

When weighted, only 6.8% (standard error, se < 0.001) of the observations were CVD/MI-positive. About 51.5% (se = 0.002) were estimated to be female, and 62.8% (se = 0.002) were estimated to be white non-Hispanic. The mean BMI was 28.13% (se = 0.0198). An estimated 30.8% (se = 0.002) of the individuals had 1–3 years of college. Only 6.5% (se < 0.001) of the population was estimated to occupy rural areas, and the mode estimated income was greater than $75,000 (47.2%, se = 0.002). About 96.6% (se < 0.001) did not use chewing tobacco or snuff products. Most of the population was estimated to be employed for wages (48.9%, se = 0.002). The estimate for depression was 18.2% (se = 0.001). The average number of days missed in the last 30 due to mental issues was estimated to be 4.0469 (se = 0.026), and the number of drinks consumed per day was 0.331 (se = 0.007). About 75.4% engaged in physical activity in the past 30 days. Table 1, Table 2, Table 3, Table 4, Table 5 and Table 6 provide the weighted distributions for income, race, education, employment status, age, and smoking status, respectively. 

### 3.2. Inferential Statistics

GLM regression with the quasi-binomial error term identified behavioral, socio-economic, psychological, and biological variables associated with CVD/MI. Table 7 has the complete model with associated odds ratios and confidence intervals. 

#### 3.2.1. Behavioral 

Those who never smoked were much less likely to have CVD/MI, odds ratio (OR) = 0.688, 95% CI = (0.623, 0.761), t = −7.296, *p* < 0.001), whereas chewing/snuff use had no additional risk even when evaluated outside the GLM. The number of drinks per week had no effect, an interesting finding that will be discussed later. Higher BMI was associated with higher risk of CVD/MI (OR = 1.023, 95% CI = (1.019, 1.027), t = 10.337, *p* < 0.001), while higher physical activity was associated with lower risk (OR = 0.842, 95% CI = (0.794, 0.893), t = −5.73, *p* < 0.001). 

#### 3.2.2. Socio-Economic

Higher incomes were associated with lower risk of CVD/MI. Those in the highest income groups had an odds ratio of 0.670 (95% CI = (0.585, 0.768), t = −5.758, *p* < 0.001). Education had no bearing on the presence of CVD, whereas employment status outside of a traditional job increased the risk. Those unable to work were (as expected) more likely to have CVD/MI (OR: 2.834, 95% CI = (2.565, 3.131), t = 19.219, *p* < 0.001), and all others that were not “employed for wages”, except for students, had higher risk. Urban residents were at lower risk than rural residents (OR = 0.886, 95% CI = (0.823, 0.953), t = −3.324, *p* < 0.001)

#### 3.2.3. Psychological

Depression was a risk factor for CVD/MI (OR = 1.505, 95% CI = (1.407, 1.609), t = 11.940, *p* < 0.001). Additionally, the number of days an individual was unable to work because of mental issues increased the risk of CVD/MI (OR = 1.013, 95% CI = (1.009, 1.015), t = 7.557, *p* < 0.001). 

#### 3.2.4. Biological

Every increase in age category increased the risk of CVD presence. For 80-year-old individuals, the OR was 26.809 (95%CI = (18.943, 37.659), t = 18.745, *p* <0.001). Males were more likely than females to have CVD (OR = 1.941, 95% CI = (1.834, 2.054), t = 22.919, *p* < 0.001). Surprisingly, only indigenous Americans, Alaskan non-Hispanics, and other race Hispanics were more at risk for CVD/MI with odds ratios of 1.276 (95% CI = (1.083, 1.503)) and 1.285 (95% CI = (1.119, 1.477)), respectively. While black non-Hispanics are known to be at higher risk for CVD and MI, this analysis indicates that black non-Hispanics are actually less likely to have CVD with an OR of 0.850 (95% CI = (0.775, 0.933)). 

## 4. Discussion

### 4.1. Behavioral Factors

As expected, behavioral, socio-economic, psychological, and biological variables affect the risk of CVD/MI. Non-smokers were associated with a reduced risk of CVD/MI. This finding is congruent with previous research [24]. Drinking was not associated with reduced CVD/MI, contrary to some prior research. There are conflicting studies about the effects of drinking on CVD/MI. Some studies have shown an effect of drinking on CVD/MI [25], while others have found no such association [26]. This study’s findings support no relationship between drinking and CVD/MI. More research is needed specifically to identify the risks of drinking and CVD/MI. 

### 4.2. Socio-Economic Factors

Socio-economic risks included income status (where higher income was associated with reduced risk) and employment status (where those unable to work were associated with the highest risk). Income is associated with better access to care [16]. The results make sense, as those with higher incomes are likely to have access to better health care (preventive and otherwise). Having access to preventive care (e.g., hypertension medicine) likely reduces risk. 

### 4.3. Psychological Factors

The effects of psychological factors are interesting. Both a history of depression and days lost due to mental issues were significant risk criteria for CVD/MI. This finding is congruent with previous studies as well [15]. Depression and days lost are likely to be risk factors, as stress may cause heart issues such as CVD/MI, which exercise may help counteract [27].

### 4.4. Biological Factors

Age and race were important biological considerations for risk of CVD/MI. Increases in age increase the risk in nonlinear fashion (see Appendix A), while the highest-risk race category was other, non-Hispanic. Race, age, and ethnicity differences make sense given previous research [28]. The directionality of the results associated with age is also reasonable given that increased age results in increased risk. Biological factors are likely to have synergistic effects when combined, and additional studies should focus on these using non-behavioral data. 

The question remains, why did Black non-Hispanics not have increased rates of CVD/MI as found in some other studies? The researchers found the results of this analysis to be contrary to the past findings, so the unweighted data were analyzed in Table 8. Those data supported the findings of the weighted analysis. Further, an unweighted analysis of all states and territories supports these findings, with only four exceeding the overall rate of CVD/MI (Table 9). Possible reasons for these findings are discussed later. Even sudden cardiac death (SCD) is not fully explained by other factors such as demographics, socioeconomics, cardiovascular risk factors, and behavioral measures [29]. It is possible that this finding is a result of under-reporting in the BRFSS, as this is incongruent with most previous analyses. Interestingly, however, is the fact that Whites engage in a most unhealthy lifestyle, while the Black population engages in an unhealthy lifestyle. In other words, Whites are higher at risk due to their behaviors [30]. This may be the reason for the findings in this study, although further inquiry is required. 

## 5. Conclusions

The strength and relevance of this study is both its recency and the sample size. It is the first such study to use the BRFSS data from 2018 for this purpose. Overall, behavioral, socio-economic, psychological, and biological variables combine for a fairly comprehensive look at the risk of CVD/MI. However, several limitations exist in this study. First, the CDC’s BRFSS is self-reported data only. While the BRFSS is based on self-reporting, the study is widely used for analysis. The prevalence rates of heart disease according to the CDC (6.7%, [31]) are similar to those of the BRFSS (6.9%). There is some slight over-representation or undiagnosed CVD. For this study, the rate of depression was 18.23%. This is similar to the 18.1% reported by the Anxiety and Depression Association of America [32]. In balance, the self-reporting appears to be at least a reasonable representation of reality. Still, the possibility of under-reporting or over-reporting exists. Second, the study focuses solely on the United States. It should not be generalized outside of this country. 

A strategy to address the disparities associated with CVD/MI is dissemination of information. Education has the potential of addressing behavioral and psychological components for the underserved population. Local interventions might include increasing access to free or discounted care at local clinics in disadvantaged socio-economic communities. Though some such programs already do exist, expansion and marketing of services as well as increasing quality are all issues for local governments to consider. 

Two ethical principles that relate to any policy solutions are the principle of beneficence and the principle of justice. The principle of beneficence is that health care providers have a duty to perform acts that benefit the patients and can assist in improving their health status [33]. This principle, when applied, can help to improve the health of lower-economic-status population and communities that do not receive the same level of health care as population with higher level of incomes. The principle of justice is defined as the ability for health care to be equal and fair for all [33]. These principles should be part of policy decision-making for addressing healthcare disparities.

## Figures and Tables

**Table 1:** Variable INCOME2: “Is your annual household income from all sources: (If respondent refuses at any income level, code “Refused”) [20]”.

Income	Proportion	Standard Error
<$10K	0.044	0.001
$10K ≤ Income < $15K	0.040	0.001
$15K ≤ Income < $20K	0.058	0.001
$20K ≤ Income < $25K	0.073	0.001
$25K ≤ Income < $35K	0.083	0.001
$35K ≤ Income < $50K	0.105	0.001
$50K ≤ Income < $75K	0.124	0.001
$75K or more	0.472	0.002

**Table 2:** Variable EDUCA: “What is the highest grade or year of school you completed? [20]”.

Grade/Year	Proportion	Standard Error
None or Only Kindergarten	0.003	0.000
Grades 1 through 8	0.044	0.001
Grades 9 through 11	0.084	0.001
Grades 12 or GED	0.278	0.002
College 1 to 3 years	0.308	0.002
College 4+ years (Graduate)	0.283	0.001

**Table 3:** Variable EMPLOY1: “Are you currently…? [20]”.

Employed Status	Proportion	Standard Error
Employed for Wages	0.489	0.002
Self-Employed	0.094	0.001
Out of Work ≥ 1 Year	0.024	0.001
Out of Work < 1 Year	0.025	0.001
Homemaker	0.058	0.001
Student	0.055	0.001
Retired	0.184	0.001
Unable to Work	0.071	0.001

**Table 4:** Variable AGE5YR: “Fourteen-Level Age Category [20]”.

Age Group	Proportion	Standard Error
18 to 24	0.123	0.001
25 to 29	0.081	0.001
30 to 34	0.092	0.001
35 to 39	0.078	0.001
40 to 44	0.081	0.001
45 to 49	0.070	0.001
50 to 54	0.086	0.001
55 to 59	0.080	0.001
60 to 64	0.085	0.001
65 to 69	0.083	0.001
70 to 74	0.056	0.001
75 to 79	0.039	0.001
80 or older	0.046	0.001

**Table 5:** Variable IMPRACE: “Imputed race/ethnicity value (This value is the reported race/ethnicity or an imputed race/ethnicity, if the respondent refused to give a race/ethnicity. The value of the imputed race/ethnicity will be the most common race/ethnicity response for that region of the state) [20]”.

Race/Ethnicity	Proportion	Standard Error
White	0.628	0.002
Black	0.117	0.001
Asian	0.054	0.001
American Indian/Alaskan	0.011	0.000
Hispanic	0.170	0.002
Other non-Hispanic	0.021	0.000

**Table 6:** Variable SMOKER3: “Four-level smoker status: Everyday smoker, Someday smoker, Former smoker, Non-smoker [20]”.

Smoking Status	Proportion	Standard Error
Smokes Every Day	0.102	0.001
Smokes Some Days	0.045	0.001
Former Smoker	0.230	0.001
Never Smoked	0.622	0.002

**Table 7:** Complete model with associated odds ratios and confidence intervals.

Variable	Estimate	S.E.	t-Value	Pr (>|t|)	Odds Ratio	95% CI Lower	95% CI Upper
Intercept	−4.945	0.534	−9.264	<0.001	0.007	0.003	0.020
Smokes Some	0.127	0.083	1.529	0.126	1.135	0.965	1.335
Former Smoker	−0.002	0.050	−0.039	0.969	0.998	0.905	1.101
Never Smoked	−0.374	0.051	−7.296	0.000	0.688	0.623	0.761
Chewing Tobacco or Snuff?	0.117	0.076	1.533	0.125	1.124	0.968	1.304
Drinks per Week	0.000	0.000	−1.618	0.106	1.000	1.000	1.000
Body Mass Index	0.023	0.002	10.337	<0.001	1.023	1.019	1.027
Exercised in 30 Days?	−0.172	0.030	−5.730	0.000	0.842	0.794	0.893
Income $[10,15)K	−0.103	0.078	−1.321	0.186	0.902	0.775	1.051
Income $[15,20)K	−0.014	0.078	−0.183	0.855	0.986	0.846	1.149
Income $[20,25)K	−0.122	0.081	−1.517	0.129	0.885	0.755	1.036
Income $[25,35)K	−0.201	0.080	−2.518	0.012	0.818	0.699	0.956
Income $[35,50)K	−0.247	0.079	−3.123	0.002	0.781	0.669	0.912
Income $[50,75)K	−0.297	0.075	−3.940	0.000	0.743	0.641	0.861
Income >$75K	−0.400	0.070	−5.758	0.000	0.670	0.585	0.768
Grades 1–8	−0.354	0.465	−0.760	0.447	0.702	0.282	1.747
Grades 9–11	−0.194	0.472	−0.410	0.682	0.824	0.327	2.077
High School Grad./GED	−0.433	0.473	−0.915	0.360	0.649	0.257	1.639
College 1 to 3 Years	−0.392	0.473	−0.828	0.408	0.676	0.267	1.709
College 4+ Year/Graduate	−0.612	0.473	−1.294	0.196	0.542	0.215	1.370
Self-Employed	0.163	0.066	2.477	0.013	1.177	1.034	1.338
Out of Work ≥ 1 Year	0.452	0.085	5.334	0.000	1.571	1.331	1.855
Out of Work < 1 Year	0.304	0.126	2.408	0.016	1.356	1.058	1.737
Homemaker	0.288	0.078	3.687	0.000	1.334	1.144	1.554
Student	0.138	0.321	0.430	0.668	1.148	0.612	2.151
Retired	0.490	0.047	10.518	<0.001	1.632	1.490	1.788
Unable to Work	0.987	0.051	19.219	<0.001	2.683	2.426	2.967
Urban?	−0.121	0.037	−3.234	0.001	0.886	0.823	0.953
Depression?	0.409	0.034	11.940	<0.001	1.505	1.407	1.609
Missed Work/Mental	0.012	0.002	7.557	0.000	1.012	1.009	1.015
Age 25–29	0.339	0.200	1.700	0.089	1.404	0.949	2.075
Age 30–34	0.615	0.221	2.784	0.005	1.849	1.200	2.849
Age 35–39	0.829	0.198	4.184	0.000	2.291	1.554	3.378
Age 40–44	1.280	0.179	7.155	0.000	3.597	2.533	5.107
Age 45–49	1.389	0.176	7.896	0.000	4.011	2.841	5.662
Age 50–54	1.794	0.169	10.592	<0.001	6.013	4.315	8.381
Age 55–59	2.077	0.170	12.240	<0.001	7.980	5.722	11.130
Age 60–64	2.420	0.170	14.235	<0.001	11.246	8.059	15.693
Age 65–69	2.596	0.170	15.299	<0.001	13.410	9.616	18.702
Age 70–74	2.898	0.173	16.734	<0.001	18.138	12.917	25.469
Age 75–79	3.118	0.176	17.700	<0.001	22.601	16.004	31.917
Age 80 or Older	3.285	0.175	18.745	<0.001	26.709	18.943	37.659
Male	0.663	0.029	22.919	<0.001	1.941	1.834	2.054
Black Non-Hispanic	−0.162	0.047	−3.416	0.001	0.850	0.775	0.933
Asian Non-Hispanic	−0.017	0.122	−0.142	0.887	0.983	0.773	1.249
American Indian/Alaskan	0.244	0.084	2.918	0.004	1.276	1.083	1.503
Hispanic	0.018	0.067	0.271	0.786	1.018	0.893	1.161
Other Race, Hispanic	0.251	0.071	3.545	0.000	1.285	1.119	1.477

**Table 8:** Evaluation of Race and cardiovascular disease/myocardial infarction (CVD/MI).

Race	No CVD/MI	CVD/MI	% CVD/MI
White, Non-Hispanic	298,046	31,868	10.69%
Black, Non-Hispanic	33,433	3010	9.00%
Asian, Non-Hispanic	10,347	537	5.19%
American Indian/Alaskan	7509	1025	13.65%
Hispanic	35,084	2235	6.37%
Other Race, Non-Hispanic	12,846	1496	11.65%

**Table 9:** CVD/MI by state and territory, where green coloring indicates lower than the total percent.

State	White	Black	Asian	Am. Indian/AL Native	Hispanic	Other	Total
Alabama	12.75%	10.44%	4.44%	23.08%	9.78%	12.23%	12.17%
Alaska	7.58%	5.77%	4.62%	10.00%	4.67%	6.29%	7.63%
Arizona	11.14%	6.47%	7.02%	5.69%	6.68%	10.33%	9.84%
Arkansas	15.60%	10.90%	0.00%	19.70%	7.32%	25.29%	14.92%
California	6.48%	6.80%	3.28%	16.52%	4.84%	6.70%	5.78%
Colorado	6.88%	8.80%	8.04%	8.97%	4.57%	7.28%	6.63%
Connecticut	8.59%	5.24%	5.11%	9.23%	4.85%	7.17%	7.96%
Delware	10.67%	6.72%	5.30%	10.14%	2.79%	9.77%	9.14%
District of Columbia	5.34%	8.63%	1.74%	11.59%	2.72%	6.00%	6.74%
Florida	12.65%	9.26%	5.69%	17.51%	6.60%	13.39%	11.65%
Georgia	10.17%	7.61%	3.39%	12.15%	3.58%	11.69%	8.84%
Hawaii	7.08%	1.09%	6.18%	11.76%	5.88%	7.78%	6.86%
Idaho	8.58%	5.56%	2.94%	18.18%	5.64%	17.95%	8.64%
Illinois	8.20%	7.67%	3.54%	10.87%	4.59%	7.45%	7.43%
Indiana	11.20%	9.66%	5.75%	11.88%	5.81%	13.33%	10.91%
Iowa	8.36%	7.69%	4.17%	18.00%	3.23%	10.20%	8.16%
Kansas	9.54%	10.36%	6.29%	13.57%	4.26%	8.29%	9.31%
Kentucky	12.70%	12.73%	2.44%	15.56%	8.94%	18.81%	12.77%
Louisiana	11.26%	7.85%	5.56%	6.49%	7.59%	9.03%	10.12%
Maine	10.99%	14.29%	10.42%	23.21%	9.52%	15.45%	11.21%
Maryland	10.13%	7.09%	6.47%	13.49%	4.41%	10.04%	9.28%
Massachusetts	7.82%	5.67%	3.06%	21.31%	5.21%	8.38%	7.48%
Michigan	9.72%	8.19%	1.73%	17.07%	7.47%	7.14%	9.38%
Minnesota	7.17%	6.63%	2.13%	11.11%	2.81%	6.89%	6.91%
Mississippi	12.19%	9.68%	15.38%	20.93%	3.92%	10.42%	11.19%
Missouri	11.63%	10.80%	5.45%	11.11%	10.62%	12.08%	11.48%
Montana	8.35%	7.27%	19.23%	12.52%	10.00%	13.75%	9.05%
Nebraska	9.10%	4.51%	6.02%	12.83%	3.83%	8.39%	8.69%
Nevada	10.46%	8.57%	4.21%	8.20%	5.38%	11.58%	9.32%
New Hampshire	9.38%	5.36%	9.76%	10.00%	6.17%	8.70%	9.29%
New Jersey	8.21%	7.97%	3.80%	13.33%	5.58%	7.04%	7.58%
New Mexico	9.92%	9.57%	3.13%	6.36%	6.48%	11.36%	8.42%
New York	9.37%	5.75%	5.17%	15.07%	5.96%	12.04%	8.88%
North Carolina	10.66%	7.88%	1.69%	18.75%	2.89%	19.82%	9.78%
North Dakota	10.64%	6.10%	5.13%	15.82%	5.56%	9.09%	10.61%
Ohio	11.26%	9.85%	4.86%	20.51%	9.48%	16.48%	11.27%
Oklahoma	13.24%	8.48%	4.76%	8.57%	5.41%	16.85%	12.31%
Oregon	7.88%	5.63%	6.25%	6.35%	5.92%	10.51%	7.77%
Pennsylvania	8.28%	7.92%	0.67%	2.78%	7.45%	6.34%	7.94%
Rhode Island	9.52%	7.04%	8.65%	15.79%	5.79%	7.65%	9.11%
South Carolina	11.57%	9.92%	3.16%	16.67%	4.11%	15.00%	10.98%
South Dakota	10.18%	12.73%	0.00%	13.24%	6.52%	10.37%	10.53%
Tennessee	11.59%	11.26%	10.53%	17.31%	5.08%	14.86%	11.55%
Texas	11.35%	9.42%	5.49%	14.67%	6.34%	15.41%	10.11%
Utah	5.73%	3.90%	1.72%	7.05%	2.83%	5.82%	5.44%
Vermont	7.44%	3.13%	2.56%	21.28%	5.88%	9.63%	7.64%
Virginia	9.66%	6.67%	5.56%	11.21%	5.25%	11.36%	8.89%
Washington	9.13%	7.14%	4.12%	9.71%	3.91%	7.88%	8.50%
West Virginia	15.04%	11.82%	13.79%	25.00%	10.42%	21.90%	15.12%
Wisconsin	7.89%	8.29%	4.35%	10.20%	4.29%	8.00%	7.83%
Wyoming	8.73%	21.43%	7.41%	16.67%	5.22%	11.76%	8.70%
Guam	3.13%	4.55%	6.15%		14.29%	10.27%	8.50%
Puerto Rico	13.04%	0.00%	0.00%	0.00%	10.70%	7.69%	10.69%
